# Effect of Meal Texture on Postprandial Glucose Excursions and Gut Hormones After Roux-en-Y Gastric Bypass and Sleeve Gastrectomy

**DOI:** 10.3389/fnut.2022.889710

**Published:** 2022-04-29

**Authors:** Nora Hedbäck, Morten Hindsø, Kirstine N. Bojsen-Møller, Adelaide K. Linddal, Nils B. Jørgensen, Carsten Dirksen, Andreas Møller, Viggo B. Kristiansen, Bolette Hartmann, Jens J. Holst, Maria S. Svane, Sten Madsbad

**Affiliations:** ^1^Department of Endocrinology, Hvidovre Hospital, Hvidovre, Denmark; ^2^Faculty of Health Sciences, Biomedical Institute, University of Copenhagen, Copenhagen, Denmark; ^3^Department of Surgery, Hvidovre Hospital, Hvidovre, Denmark; ^4^Novo Nordisk Foundation Center for Basic Metabolic Research, University of Copenhagen, Copenhagen, Denmark

**Keywords:** bariatric surgery, liquid meal, solid meal, glucagon-like peptide-1, insulin secretion, ghrelin

## Abstract

**Background and aims:**

The metabolic consequences after Roux-en-Y gastric bypass (RYGB) and sleeve gastrectomy (SG) are often studied using a liquid mixed meal. However, liquid meals may not be representative of the patients’ everyday diet. We therefore examined postprandial glucose and gut hormone responses using mixed meals differing only with respect to meal texture.

**Methods:**

Twelve RYGB-operated, 12 SG-operated, and 12 unoperated individuals (controls) were enrolled in the study. Participants were matched on age, sex, and body mass index. In randomized order, each participant underwent a liquid and a solid 4-h mixed meal test on separate days. The meals were isocaloric (309 kcal), and with identical macronutrient composition (47 E% carbohydrate, 18 E% protein, 32 E% fat, and 3 E% dietary fibers). The liquid meal was blended to create a smooth liquid texture while the other meal retained its solid components.

**Results:**

Postprandial glucose concentrations (peak and incremental area under curve, iAUC) did not differ between the two meal textures in any group. In the control group, peak C-peptide was higher after the liquid meal compared with the solid meal (*p* = 0.04), whereas iAUCs of C-peptide were similar between the two meals in all groups. Peak of glucagon-like peptide-1 (GLP-1) was higher after the liquid meal compared with the solid meal in RYGB- and SG-operated individuals (RYGB *p* = 0.02; SG *p* < 0.01), but iAUC of GLP-1 did not differ between meal textures within any group. Peak of glucose-dependent insulin tropic polypeptide (GIP) was higher after the liquid meal in the SG and control groups (SG *p* = 0.02; controls *p* < 0.01), but iAUCs of GIP were equal between meals. There were no differences in total AUC of ghrelin between the liquid and solid meals within any of the groups.

**Conclusion:**

A liquid and a solid meal with identical macronutrient composition result in similar postprandial glucose responses, both in operated and unoperated individuals. Small differences were observed for the postprandial peaks of C-peptide, GLP-1, and GIP concentrations. Overall, a liquid meal is suitable for evaluating glucose tolerance, β-cell function, and gut hormones responses, both after RYGB and SG and in unoperated individuals.

**Clinical Trial Registration:**

[www.clinicaltrials.gov], identifier [NCT04082923].

## Introduction

Bariatric surgery leads to significant changes in body weight and results in improvements of obesity-related metabolic disorders including type 2 diabetes (1). Roux-en-Y gastric bypass (RYGB) and sleeve gastrectomy (SG) both result in good metabolic outcomes despite diverging surgical techniques (1). While the upper gastrointestinal tract is rearranged after RYGB with a small gastric pouch anastomosed directly to the distal portion of the jejunum excluding food from the stomach, duodenum, and proximal jejunum (2), the SG procedure consists of a longitudinal excision of the greater curvature of the stomach leaving a narrow tube for nutrient passage to the duodenum (3).

The modified nutrient delivery to the intestines and the altered gut hormone secretion are of paramount importance for the weight loss and diabetes remission after these two surgical procedures (4–6). The rate of nutrient delivery to the small intestines and the small intestinal transit is important for the release of pancreatic and gut hormones including the incretin hormones, glucagon-like peptide-1 (GLP-1), and glucose-dependent insulin tropic polypeptide (GIP) (5, 6). Previous studies using liquid meals have indicated that nutrient transit and absorption are faster after RYGB compared with SG and unoperated individuals resulting in greater glucose excursions and GLP-1 responses (5–7). The increased GLP-1 secretion after RYGB stimulates postprandial insulin secretion and improves glucose tolerance (8–11). After SG, the release of GIP appears to be larger compared with RYGB, and the orexigenic gut hormone ghrelin decreases drastically, which may be of importance for the weight loss after this procedure (12–14). Studies have demonstrated longer transit time through the small intestines for solid meals compared with liquid meals after RYGB (5, 15), while intestinal transit of solid and liquid nutrients have been reported to be comparable in unoperated individuals (16).

The metabolic consequences of RYGB and SG are often studied using mixed meal tests, providing a physiological stimulation of glucose metabolism and gut hormone release. However, mixed meal tests are not standardized. Several studies have used either commercial or *ad hoc* produced liquid mixed meals with diverging composition and sources of macronutrients, while more everyday meals with solid food components are used in other studies (6, 17–19). If food texture affects postprandial glucose metabolism and pancreatic and gut hormone release after RYGB and SG, the use of liquid meals in many previous studies could have affected outcomes. We hypothesize that a liquid mixed meal would induce a higher postprandial GLP-1 response compared with a solid meal both in RYGB and SG operated individuals.

We therefore investigated the importance of meal texture for postprandial glucose metabolism and gut hormone responses after RYGB and SG and in unoperated individuals using meals with identical composition, differing only with respect to texture.

## Materials and Methods

### Participants

Twelve RYGB- and 12 SG-operated individuals who had undergone bariatric surgery more than 12 months before at Hvidovre University Hospital, Denmark, were included. The operated participants were individually matched on age, sex, and body mass index (BMI) both before and after surgery. In addition, 12 unoperated controls were matched on age, sex, and BMI. All individuals were weight-stable (±3 kg during the last month) and did not have a history of type 2 diabetes (HbA1c < 48 mmol/mol pre- and postoperatively). The exclusion criteria were unstable thyroid disease, serious heart or respiratory illness, hemoglobin < 6.5 mmol/L, pregnancy, or breastfeeding.

### Ethics

The study was approved by the Regional Ethical Committee of the Capital Region (Protocol number: H-19027100) and by the Danish Data Protection Agency. It was performed in accordance with the Helsinki declaration and the study was registered at ClinicalTrials.gov (NCT04082923). Before inclusion, written informed consent was obtained from all participants.

### Study Design

On two separate study days, each participant underwent a solid and a liquid 4-h mixed meal test performed in a randomized order. The two study visits were performed at least 2 days apart. On the study days, the participants arrived after a minimum 10-h overnight fast. Anthropometric measures were obtained, and participants were placed in a reclined position in a hospital bed and no physical activity was allowed throughout the test day. An intravenous catheter was inserted into an antecubital vein and three baseline blood samples were drawn (at −10, −5, and 0 min). The meals were served at time 0 min and consisted of 65 g boiled potato, 40 g roasted chicken breast, 9 g raisins, 40 g pineapple, 65 g Patak’s^®^ butter chicken sauce (Patak’s^®^, Leigh, Great Britain), 1.75 g grated coconut, 1.5 g psyllium seeds and 100 g milk (0.4 fat E%) comprising a total of 309 kcal (47 E% carbohydrate, 18 E% protein, 32 E% fat and 3 E% dietary fibers). The ingredients of the solid meal were served, thoroughly mixed, in its original textures while the glass of milk served separately. The liquid meal was prepared by blending the solid food components with the milk (using OBH Nordica Ultimate Compact 6830 blender (OBH Nordica Denmark A/S). All test meals were prepared by the same investigator in the morning of each study day. The composition and energy content of the two test meals were identical and were composed by a clinical dietitian. The meals followed the “Nordic Nutrition Recommendation” (20). To measure absorption rate, 1 g of crushed paracetamol (Pamol, Nycomed, Roskilde, Denmark) was added to the first tablespoon of the liquid meal as a surrogate estimate of the intestinal nutrition exposure. Both meals were consumed evenly over 20 min under close supervision (including the glass of milk at the solid meal day, where intake also was distributed during the 20 min). Blood was sampled frequently for 4 h after initiation of the meal (At time 5, 10, 15, 20, 25, 30, 45, 60, 90, 120, 150, 180, and 240 min).

### Sample Collection and Laboratory Analyses

Blood was collected into pre-chilled Ethylenediamine tetraacetic acid (EDTA)-coated tubes for plasma glucose analysis, centrifuged at room temperature for 45 s and analyzed bedside using the glucose oxidase method (YSI 2300 STAT Plus; YSI, Yellow Springs, OH, United States). Samples for serum C-peptide and paracetamol analyses were collected in clot activator tubes and left to coagulate at room temperature before centrifugation at 4°C for 10 min. Further blood samples were collected into pre-chilled EDTA tubes containing a specific dipeptidyl peptidase 4 (valine-pyrrolidide, final concentration 0.01 mM, a gift from Novo Nordisk, Bagsværd, Denmark) for the analysis of GLP-1 and GIP. EDTA tubes were centrifuged immediately at 4°C for 10 min. Serum was stored at −80°C and plasma at −20°C until batch analysis.

Serum C-peptide concentrations were determined by Immulite 2000 analyzer (Siemens Healthcare Diagnostics, Tarrytown, NY, United States). Paracetamol concentrations, from the liquid test day only, were analyzed using reagents from SEKISUI^®^ Diagnostics (Abbott, Denmark) (21). Total GLP-1 and total GIP were measured by radioimmunoassays as previous described (22–24). Total ghrelin was measured using a Millipore ELISA kit (cat. no. EZGRT-89K, Billerica, MA, United States) (25).

### Calculations

Fasting concentrations were calculated as the mean of two (and for plasma glucose, three) basal blood samples. Total area under the curve (tAUC) was calculated using the trapezoidal rule and incremental AUC (iAUC) with subtracted fasting concentration. The primary outcome was the difference in tAUC of GLP-1 between the liquid and solid meals (ΔtAUC GLP-1_Liq–Sol_) comparing between the three groups individuals. Rate of intestinal nutrition exposure was estimated using time to peak of paracetamol concentrations (T_max_ pcm) as validated previously (26). The insulin secretion rates (ISRs) were derived *via* deconvolution of peripheral C-peptide concentrations, as described previously (27). β-Cell glucose sensitivity was calculated as the slope of the linear relation between ISR and the corresponding plasma glucose concentration from fasting to peak of plasma glucose for each patient (28). β-GS is an index demonstrating the dynamic changes in insulin secretion in response to glucose as modified by changes in incretin hormones, other nutrients, and neuronal inputs during the meals.

Two-tailed, paired Student’s *t*-test was applied to test for within-group differences between the liquid and solid meals. For each meal test (liquid and solid), between-group differences were evaluated with one-way ANOVA, followed by a *post hoc* Tukey’s test to correct for multiple comparisons. Logarithmic transformation was used in case of a skewed distribution, and a Shapiro–Wilk test was used to test for normal distribution of residuals. Statistical analyses were performed in R, version i386 4.0.2^1^. A value of *p* < 0.05 was considered significant. All values are mean ± SEM, unless otherwise stated.

## Results

### Baseline Characteristics

Twelve SG-operated, 12 RYGB-operated, and 12 unoperated control participants were included. The groups were individually matched on age (mean ± SD; RYGB 50.7 ± 8.1 years, SG 52.4 ± 9.0, control 51.1 ± 9.9), sex (8 women in each group), and BMI (RYGB 31.9 ± 1.4, SG 32.4 ± 1.2, control 31.6 ± 1.1). In addition, the surgical groups were matched in terms of preoperative BMI (RYGB 42.9 ± 1.5, SG 41.9 ± 1.3). There was no difference in time since surgery between the two operated groups (median [interquartile range); RYGB 1.4 (0.95) years, SG 1.4 (0.45)]. The two study visits were performed at 5.9 ± 0.9 days apart.

### Glucose

**FIGURE 1:**
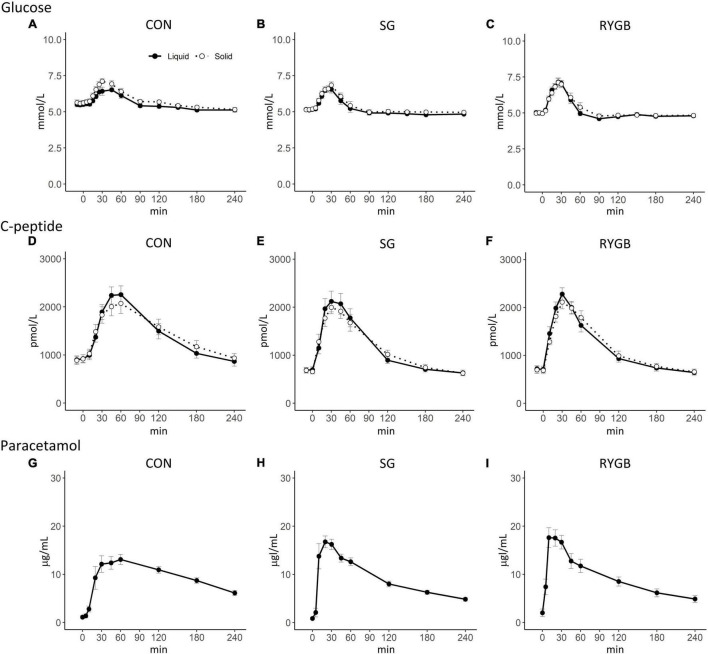
Plasma concentrations of plasma glucose and C-peptide on two different study days with either a liquid or a solid meal. Plasma concentration of and paracetamol was studied after a liquid meal. **(A–C)** Plasma glucose **(D–F)** C-peptide **(G–I)** Paracetamol. Data are means ± SEM. Black (solid line): liquid; white (dotted line): solid.

The two meal textures did not cause differences in peak, iAUC, nadir, or time to peak of plasma glucose concentrations in any of the three groups (Table 1 and Figure 1).

**TABLE 1:** Postprandial glucose, C-peptide, and paracetamol concentrations on the two test days, liquid or solid meal.

		CON, mean ± SEM	SG, mean ± SEM	RYGB, mean ± SEM	*p*-value ANOVA	CON vs SG	CON vs RYGB	SG vs RYGB
**Glucose**								
Fasting, *mmol/L*	Liq	5.5 ± 0.1	5.2 ± 0.1	5.0 ± 0.1	0.01^a^	0.11	0.01	0.59
	Sol	5.6 ± 0.2	5.2 ± 0.06	5.0 ± 0.1	< 0.01^a^	0.04	< 0.01	0.51
Peak, *mmol/L*^b^**	Liq	6.9 ± 0.2	6.9 ± 0.2	7.4 ± 0.2	0.13	−	−	−
	Sol	7.3 ± 0.2	7.0 ± 0.2	7.4 ± 0.3	0.43	−	−	−
iAUC *mmol/L × min*	Liq	−181 ± 22	−178 ± 15	−147 ± 17	0.36	−	−	−
	Sol	−157 ± 23	−149 ± 13	−124 ± 18	0.43	−	−	−
Nadir PG, *mmol/L*^b^**	Liq	4.8 ± 0.1	4.5 ± 0.1	4.4 ± 0.1	< 0.01	0.01	< 0.01	0.94
	Sol	5.1 ± 0.1	4.8 ± 0.1	4.5 ± 0.2	< 0.01	0.09	< 0.01	0.46
Time to peak, *min^b^*	Liq	34.2 ± 4.3	23.8 ± 1.6	21.3 ± 1.8	0.01	0.03	0.02	0.31
	Sol	29.6 ± 3.6	25.4 ± 1.7	25.0 ± 2.5	0.56	−	−	−
**C-peptide**								
Fasting, *pmol/L*	Liq	912 ± 81	692 ± 59	712 ± 67	0.06	−	−	−
	Sol	904 ± 85	672 ± 49	693 ± 71	0.05	−	−	−
Peak, *pmol/L*	Liq	2,353 ± 193	2,251 ± 200	2,365 ± 136	0.89	−	−	−
	Sol	2,136 ± 195**^+^**	2,087 ± 140	2,203 ± 150	0.88	−	−	−
iAUC, *nmol/L × min*	Liq	186 ± 19	148 ± 14	147 ± 11	0.13	−	−	−
	Sol	200 ± 22	155 ± 17	159 ± 13	0.16	−	−	−
Time to peak, min^b^	Liq	51.3 ± 2.9	33.8 ± 3.6	30.8 ± 2.2	< 0.01	<0.01	< 0.01	0.73
	Sol	52.9 ± 4.0	34.6 ± 3.4	36.3 ± 2.2	< 0.01	<0.01	< 0.01	0.52
β-cell GS pmol ⋅ kg^–1^ ⋅ min^–1^/(mmol/l)^b^	Liq	4.7 ± 0.75	5.3 ± 0.66	4.1 ± 0.52	0.38	−	−	−
	Sol	3.1 ± 0.27**^+^**	4.6 ± 0.69	3.5 ± 0.51	0.17	−	−	−
**Paracetamol**								
Time to peak, *min*	Liq	64.2 ± 10	21.7 ± 2.7	20.0 ± 2.5	< 0.01	<0.01	< 0.01	0.98

*Data are mean ± SEM. iAUC, incremental area under the curve with the subtraction of baseline concentration. β-cell GS, β-cell glucose sensitivity. Comparisons within groups were made using paired two-sample t-test. Between-group differences were evaluated with a one-way ANOVA, followed by a post hoc Tukey’s test.*

*^a^model on logarithmic data*

*^b^post hoc test. ^+^p < 0.05 liquid vs. solid.*

Fasting glucose concentrations were lower in RYGB-operated individuals compared with control individuals and were lower in SG-operated compared with control individuals before the solid meal, but there were no differences between the two surgical groups in fasting glucose concentrations. The peak concentration and iAUC of glucose were not statistically significantly different between groups. Nadir of plasma glucose was lower in RYGB after both the liquid and solid meals compared with control individuals, but only significantly lower after the liquid meal in SG compared with control individuals. Time to peak of plasma glucose was longer in the control group compared with the two operated groups after the liquid meal, whereas no difference between groups was observed after the solid meal (Table 1 and Figure 1).

### C-Peptide

The iAUC of C-peptide concentrations did not differ between textures in any group (RYGB *p* = 0.36; SG *p* = 0.16; control *p* = 0.48) (Table 1 and Figure 1). In the control group, peak C-peptide concentrations were higher during the liquid compared with the solid meal (*p* < 0.05), while there were no texture-related postprandial differences in C-peptide in the two operated groups (Table 1 and Figure 1). Fasting C-peptide concentrations tended to be highest in control individuals compared with the operated groups. There were no significant between-group differences in iAUC or peak C-peptide concentrations after the two meals.

In the control group, β-cell glucose sensitivity was increased after the liquid meal compared with the solid meal (*p* = 0.02), but there were no differences between the meals in the two operated groups. There were no differences between the groups regarding β-cell glucose sensitivity whether in response to the liquid or the solid meal.

### Gastrointestinal Hormones

#### GLP-1

The peak of GLP-1 concentrations was higher after the liquid meal compared with the solid meal in the SG and RYGB groups (SG *p* < 0.01; RYGB *p* = 0.02), while no difference between textures was seen in the control group (*p* = 0.75). tAUC and iAUC of GLP-1 after the solid and liquid meals were comparable in all groups.

**FIGURE 2:**
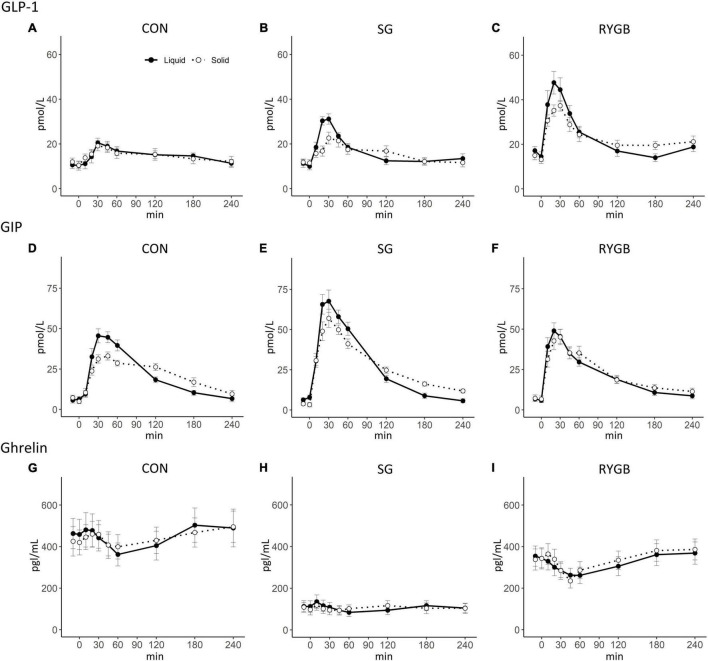
Plasma concentrations of total GLP-1, total GIP, and total ghrelin on two different study days with either a liquid or a solid meal. **(A–C)** total GLP-1; **(D–F)** total GIP; **(G–I)** total ghrelin. Data are mean ± SEM. Black (solid line), liquid; white (dotted line), solid.

The tAUC of GLP-1 concentrations was higher after RYGB compared with the SG and control group after both meals, whereas differences in iAUC of GLP-1 between the groups did not reach statistically significance. ΔtAUC GLP-1_Liq–Sol_ did not differ between the three groups (Table 2 and Figure 2).

**TABLE 2:** Postprandial gut-hormone concentrations on the two test days, liquid or solid meal.

		CON, mean ± SEM	SG, mean ± SEM	RYGB, mean ± SEM	*p*-value ANOVA	CON vs SG	CON vs RYGB	SG vs RYGB
**GLP-1**								
Fasting, *pmol/L*	Liq	10.5 ± 1.7	10.7 ± 1.6	15.8 ± 1.8	0.06	−	−	−
	Sol	11.3 ± 1.6	11.7 ± 1.3	14.1 ± 1.3	0.39	−	−	−
Peak, *pmol/L*	Liq	22.8 ± 2.3	32.7 ± 2.1	58.0 ± 6.4	< 0.01^a^	0.22	< 0.01	<0.01
	Sol	23.5 ± 2.0	26.7 ± 2.0**^++^**	42.0 ± 1.9**^+^**	< 0.01^a^	0.50	< 0.01	<0.01
tAUC, *pmol/L × min*	Liq	3,610 ± 1,138	3,863 ± 1,062	5,379 ± 1,578	< 0.01	0.88	< 0.01	0.02
	Sol	3,542 ± 1,539	3,735 ± 1,326	5,520 ± 1,595	< 0.01	0.95	< 0.01	0.02
iAUC, *pmol/L × min*	Liq	1,090 ± 270	1,303 ± 263	1,579 ± 597	0.70	−	−	−
	Sol	843 ± 137	935 ± 462	2,140 ± 501	0.08	−	−	−
ΔtAUC Liq-Sol, *pmol/L × min*		67.1 ± 470	−141 ± 508	127 ± 243	0.90	−	−	−
Time to peak, *min*^b^	Liq	35.4 ± 3.2	23.3 ± 1.4	25.4 ± 2.3	< 0.01	<0.01	0.02	0.61
	Sol	47.0 ± 12.7	34.6 ± 8.2	29.0 ± 2.5	0.32	−	−	−
**GIP**								
Fasting, *pmol/L*^b^**	Liq	6.0 ± 1.7	7.1 ± 1.2	6.4 ± 1.2	0.85	−	−	−
	Sol	6.1 ± 1.4	3.6 ± 1.1**^+^**	7.0 ± 2.0	0.28	−	−	−
Peak, *pmol/L*	Liq	49.3 ± 3.4	73.5 ± 5.7	50.4 ± 4.9	< 0.01	< 0.01	0.99	< 0.01
	Sol	36.8 ± 2.1**^+++^**	59.8 ± 5.4**^+^**	47.6 ± 4.9	< 0.01	<0.01	0.21	0.14
tAUC, *pmol/L × min*	Liq	5,096 ± 1,573	6,474 ± 1,753	5,163 ± 1,873	0.11	−	−	−
	Sol	5,211 ± 1,403	6,634 ± 1,618	5,471 ± 2,294	0.14	−	−	−
iAUC, *pmol/L × min*	Liq	3,646 ± 567	4,764 ± 508	3,633 ± 389	0.19	−	−	−
	Sol	3,751 ± 462	5,774 ± 461	3,790 ± 614	0.01	0.02	0.99	0.03
Time to peak, *min^b^*	Liq	36.3 ± 2.9	29.2 ± 4.3	22.5 ± 1.8	< 0.01	0.04	< 0.01	0.39
	Sol	52.9 ± 9.5	34.2 ± 2.4	25.0 ± 1.5	< 0.01	0.09	< 0.01	0.01
**Ghrelin**								
Fasting, *pg/mL*	Liq	461 ± 73	113 ± 27	349 ± 46	< 0.01	<0.01	0.30	< 0.01
	Sol	423 ± 65**^+^**	103 ± 23	340 ± 50	< 0.01	<0.01	0.47	< 0.01
tAUC, *ng/mL*	Liq	136 ± 78	31 ± 23	98 ± 49	< 0.01	<0.01	0.22	0.01
	Sol	137 ± 68	32 ± 24	104 ± 48	< 0.01	<0.01	0.28	< 0.01

*Data are mean ± SEM, t-AUC, total are under the curve, i-AUC, incremental area under the curve with the subtraction of baseline concentration. GLP-1, glucagon-like peptide-1; GIP, glucose-dependent insulin tropic polypeptide. Comparisons within groups were made using paired two-sample t-test. Between-group differences were evaluated with an ANOVA, followed by a post hoc Tukey’s test.*

*^a^model on logarithmic data*

*^b^post hoc test. ^+^p < 0.05 liquid vs. solid; ^++^p < 0.01 liquid vs. solid; ^+++^p < 0.001 liquid vs. solid.*

#### GIP

Peak GIP concentrations were higher after the liquid compared with the solid meal both in the SG and control groups (SG *p* = 0.02; control *p* < 0.01), whereas no difference was found in the RYGB group (*p* = 0.62). Neither tAUC nor iAUC of GIP differed between the two test days within any of the groups. Peak GIP concentration was higher in the SG group compared with the RYGB group after the liquid meal and compared with the control group after both the liquid and solid test meals. Also, after the solid meal iAUC of GIP concentrations was higher in the SG group both compared with the RYGB and control group, whereas no statistical between-group differences were seen after the liquid meal (Table 2 and Figure 2).

#### Ghrelin

There were no differences in tAUC of ghrelin between the liquid and solid meals within any of the groups. Ghrelin concentrations were markedly lower in the fasting state after SG and remained low throughout the postprandial period resulting in greatly reduced tAUC compared with both RYGB and control groups (Table 2 and Figure 2). Fasting ghrelin neither differed between RYGB and control individuals nor on test days within the groups except for a slightly higher concentration before the liquid meal in the control group.

### Gastric/Pouch Emptying

Time to the peak paracetamol concentration was longer in the control group compared with both surgical groups. No difference was found between the two operated groups (Table 1 and Figure 1). No participants experienced dumping symptoms during any meals.

## Discussion

In this study, we examined whether the texture of a mixed meal induces differences in postprandial glucose and pancreatic and gut hormone responses. This was studied in participants with previous SG and RYGB operations and in unoperated control individuals. Our primary finding was that peaks of GLP-1 concentrations were higher after the liquid meal compared with the solid meal in the RYGB- and SG-operated individuals. Furthermore, we observed a significantly increased tAUC of GLP-1 in RYGB compared with SG and control groups after both the liquid and the solid meals. Integrated GIP responses were higher after SG compared with both the RYGB group and control subjects, and were, despite higher peak responses, independent of meal texture. GIP responses did not differ between RYGB and controls. Ghrelin responses within groups were independent of meal texture, but lower in SG compared with RYGB and controls. Glucose and C-peptide responses did not differ between solid and liquid meals within the groups, but postprandial glucose nadir was lower in the RYGB-operated group compared with control individuals.

The metabolic consequences and possible differences between RYGB and SG are most frequently studied using a mixed meal test. However, as mentioned above, mixed meal tests are not standardized, and the texture of the meal could conceivably change hormone responses and thus influence conclusions regarding differences between the operations. For instance, it has previously been established that postprandial release of GLP-1 varies according to meal composition in overweight participants (29) and carbohydrate, rather than proteins or fat, has been shown to be the predominant factor responsible for the exaggerated GLP-1 and PYY responses after RYGB (30).

A study in RYGB-operated individuals showed that a liquid meal resulted in a larger GLP-1 response compared with a solid meal (18) consistent with our findings of higher early peak values. Another pilot study, with few participants (*n* = 6), evaluated the effect of meal texture for changes in GLP-1, GIP, glucose, and insulin responses in individuals after RYGB, SG, and a control group after medically induced weight loss. In that study, a liquid mixed meal caused higher GLP-1 and GIP secretion compared with a solid mixed meal in both the RYGB- and SG-operated participants (31). However, the meals were not isocaloric and differed in macronutrient composition, which is likely to have affected outcomes. As noted above, the macronutrient composition of the meal is important for the postprandial gut hormone response and glucose excursions both in unoperated individuals and especially after RYGB surgery (30). In contrast, our study using isocaloric meals with identical composition provides a more controlled method for exploration of the isolated importance of meal texture.

The most important finding of this study is that a liquid meal seems to be a valid method for studying the postprandial glucose metabolism after bariatric surgery as well as in unoperated individuals. Remarkably, meal texture neither affected peak, iAUC nor nadir plasma glucose levels or C-peptide concentrations in the two operated groups. Hence, the postprandial glucose metabolism seems to depend primarily upon macronutrient composition (30), type of carbohydrates (32), and the glucose load (33), but not meal texture.

We demonstrated higher peak GLP-1 secretion after the liquid meal in both the RYGB and SG groups compared with the solid meal. This lends credit to the notion that the GLP-1 release could in fact be modulated by meal texture. The mechanism behind the increased peak GLP-1 secretion after the liquid meal may be explained by a more rapid nutrient passage to distal intestinal segments with increasing exposure of the more distally located L-cells (34–37). We also observed an increase in peak GIP in the SG and control group after the liquid meal, which may be explained by a faster delivery of nutrient to the duodenum with the highest density of the GIP-secreting K-cells (37). However, importantly, the increased GLP-1 and GIP concentrations after liquid meal did not translate into differences in peak C-peptide concentrations or nadir of glucose concentrations. This is a central finding that can support dietetic advice for bariatric patients after surgery. Thus, it appears that meal texture alone might not have a clinical impact on bariatric operated individuals’ daily life. Notable, no participants experienced duping symptoms at neither the liquid nor solid meals.

Nevertheless, the increased early GLP-1 response after the liquid meal test day in RYGB and SG individuals might be clinically relevant in patients suffering from post-bariatric hypoglycemia (PBH). PBH is known to be caused—at least in part—by markedly increased GLP-1 secretion (38). As such, it could be hypothesized that patients suffering from PBH could experience worsening of symptoms after intake of a liquid, as opposed to a solid meal. However, in the present study, postprandial nadir glucose was lower after both liquid and solid meals in both RYGB and SG group compared with control individuals. Moreover, the study was not performed with individuals suffering from PBH. The clinical nutrient recommendation to individuals with postprandial hypoglycemia is to focus on type and content of carbohydrate content in the meal to reduce the risk of hypoglycemia (39).

We did not observe major differences in hormone responses between the textures in any groups. This may be partly explained by the design of the study as the two meals were ingested evenly over 20 min. This was chosen to standardize the intake but could also attenuate differences between textures and make the solid food components more liquid, minimizing differences between the meal in relation to gastric emptying rate and intestinal transit time. The transit rate of food delivery to the small intestines has been shown to be of major importance for the hormone release (7). Our study showed an increase in the rate of intestinal nutrient exposure in both surgical groups compared with controls. The accelerated nutrient entry in the SG and RYGB groups agrees with previous findings although the rate of entry is often faster after RYGB (5).

The study had several strengths. The order of the meals—liquid versus solid—was randomly assigned, and by design, we were able to examine patients in the weight-stable phase. In addition, the crossover design provides statistical strength. Both surgical groups were matched on both pre- and postoperative BMI and for weight loss. This minimizes the confounding effect of differing postoperative weight losses. Moreover, the meals were composed according to the healthy “Nordic nutrition recommendation” and the results are therefore directly applicable to a real-world scenario. Our results do not provide support for changes in the existing nutritional recommendations after bariatric surgery. A limitation of the study is that intestinal nutrient entry was determined by measuring paracetamol absorption. Another limitation is also that we did not measure paracetamol concentration after the solid meal, and thus we cannot evaluate gastric emptying rate after this meal. However, this technique may not be used to assess solid gastric emptying. A previous study has shown that only a small part of a paracetamol dosage remains in the solid proportion of a meal consisting of both solid and liquid components. Therefore, measuring time to peak of paracetamol concentrations after the solid meal will probably overestimate gastric emptying rate (40, 41).

## Conclusion

Liquid mixed meals are as useful as identically composed solid meals for the evaluation of glucose tolerance and β-cell response in patients after bariatric surgery. However, meal texture may affect the postprandial gut hormone response slightly after bariatric surgery, which should be considered when studying and interpreting the metabolic changes in this group of patients.

## Data Availability Statement

The raw data supporting the conclusions of this article will be made available by the authors, without undue reservation.

## Ethics Statement

The studies involving human participants were reviewed and approved by Regional Ethical Committee of the Capital Region (Protocol number: H-19027100). The patients/participants provided their written informed consent to participate in this study.

## Author Contributions

NH, MH, KB-M, NJ, CD, JH, MS, and SM contributed to the conception and design of the research. NH and AM performed the experiments. NH, MH, MS, and SM analyzed the data. BH and JH performed the hormone analysis. NH, MH, KB-M, NJ, CD, VK, BH, JH, MS, and SM interpreted the results of experiments. NH and MH prepared the figures. NH and AL designed the meals. NH, MH, MS, and SM drafted the manuscript. All authors edited, revised, and approved the final version of manuscript.

## Conflict of Interest

The authors declare that the research was conducted in the absence of any commercial or financial relationships that could be construed as a potential conflict of interest.

## Publisher’s Note

All claims expressed in this article are solely those of the authors and do not necessarily represent those of their affiliated organizations, or those of the publisher, the editors and the reviewers. Any product that may be evaluated in this article, or claim that may be made by its manufacturer, is not guaranteed or endorsed by the publisher.

## Footnotes

1
www.R-project.org
